# Short-term results of bilateral internal mammary arterial grafting for patients aged 60–75 years – a retrospective study

**DOI:** 10.1186/s13019-019-1006-8

**Published:** 2019-10-15

**Authors:** Yilong Guo, Xiaoqi Wang, Shuwu He, Yue Shu, Tianguang Wang, Zelun Chen

**Affiliations:** 1 Department of Cardiovascular Surgery, The Second Affiliated Hospital of Hainan Medical University, 48th of Bai Shui Tang Road, Haikou, Hainan People’s Republic of China 570311; 2 Department of Cardiovascular Surgery, Fuwai Hospital, Beijing, People’s Republic of China

**Keywords:** Coronary artery disease, Bilateral internal mammary artery grafting, Coronary artery bypass grafting, Sternal wound complication, Elderly patients

## Abstract

**Background:**

Bilateral internal mammary artery (BIMA) grafting has a good long-term survival rate and graft patency rate, but it is only recommended in young patients due to its high technical requirements and high incidence of sternal complications. Previous studies indicated that BIMA grafting has a significant benefit in patients aged 50–59 years, but this benefit does not extend to patients aged > 60 years. Thus, this study was designed to analyse the immediate artery graft function, short-term (3 months) results, and experience in preventing sternal complications for BIMA grafting in elderly patients (60–75 years old).

**Methods:**

Clinical records and echocardiographic and coronary artery computed tomography angiography data of 155 patients who underwent BIMA grafting for coronary artery disease between 2015 and 2017 in our hospital were analysed retrospectively to summarise the operative experience and short-term (3 months) results. Patients were divided into two groups: Group A (*n* = 95), aged < 60 years and Group B (*n* = 60), aged 60–75 years. The operation time, aortic clamp time, and cardiopulmonary bypass time of these two groups were compared to analyse the operation difficulty and the flow and pulsatility index were compared to analyse the immediate artery graft function. The left ventricular end-diastolic dimension (LVEDD) and left ventricular ejection fraction (LVEF) of these two groups were compared to analyse heart function.

**Results:**

There were no significant differences in the operation time, aortic clamp time, and cardiopulmonary bypass time as well as the flow and pulsatility index between these two groups (*P* > 0.05). There was no significant difference in the incidence of sternal wound complications, graft occlusion, and other common complications 3 months post-BIMA grafting between these two groups (*P* > 0.05). Furthermore, there was no significant difference in LVEDD and LVEF between the groups 3 months post-operation (*P* > 0.05).

**Conclusions:**

BIMA grafting was safe and effective for older patients (60–75 years). Similar to younger patients (< 60 years), BIMA grafting in elderly patients (60–75 years) can also achieve a satisfactory short-term (3 months) result. Thus, advanced age (60–75 years) should not be a contraindication for BIMA grafting.

## Background

With increasing global life expectancy and the aging population, the proportion of coronary artery bypass grafting (CABG) for elderly patients is increasing [1]. In recent years, an increasing number of scholars have focused their attention on total arterialized CABG due to its excellent patient survival rate [2, 3]. The reason for that is the high patency rate of arterial grafts, as well as the protective effect of arterial grafts on the coronary circulation of the patients [4]. At present, a large number of studies have shown that BIMA grafting is superior to single internal mammary artery (SIMA) grafting in cardiac function protection and long-term survival after surgery [5, 6]. However, due to the long operation time, high technical requirements, and high incidence rate of sternal wound complications, the application of BIMA grafting is limited [7]. At present, the application rate of BIMA grafting is about 5% worldwide, and it is only recommended for younger patients [8]. Mohammadi et al. reported that BIMA grafting has significant benefit in patients aged 50–59 years, but this benefit does not extend to patients aged > 60 years [9]. Thus, this study was designed to analyse the operation experiences, immediate artery graft function, short-term results, and experience in preventing sternal complications of BIMA grafting in elderly patients (60–75 years old).

## Methods

### Patients

From December 2015 to August 2017, 185 patients with coronary artery disease underwent BIMA grafting in our hospital. The inclusion criteria were as follows: patients with left main coronary artery plus triple-vessel disease or only triple-vessel disease. The exclusion criteria were as follows: 1) emergency surgery or patients with other severe cardiac diseases requiring concurrent surgery; 2) patients with severe heart failure or multiple organ dysfunction before the operation; and 3) preoperative computed tomography angiography (CTA) showing proximal subclavian artery or internal mammary artery stenosis. Finally, 155 patients were included in this retrospective study. The patients were divided into two groups: Group A included 95 patients, aged < 60 years and Group B included 60 patients, aged 60–75 years.

### Operative methods

**Fig. 1:**
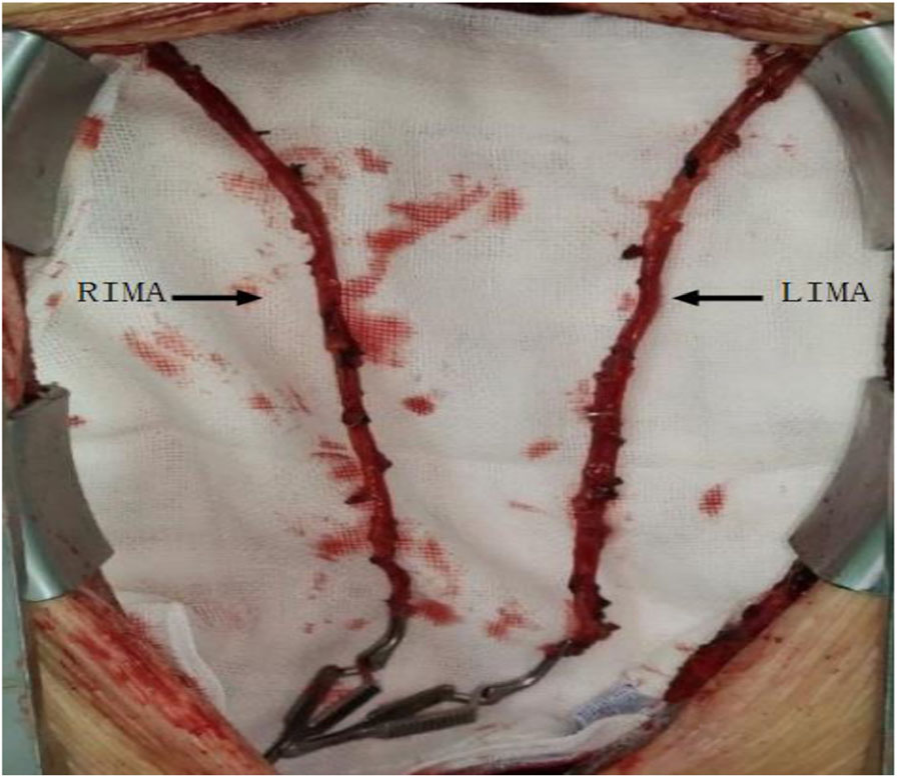
Bilateral internal mammary arteries were harvested with skeletonization technique. LIMA, left internal mammary artery; RIMA, right internal mammary artery

**Fig. 2:**
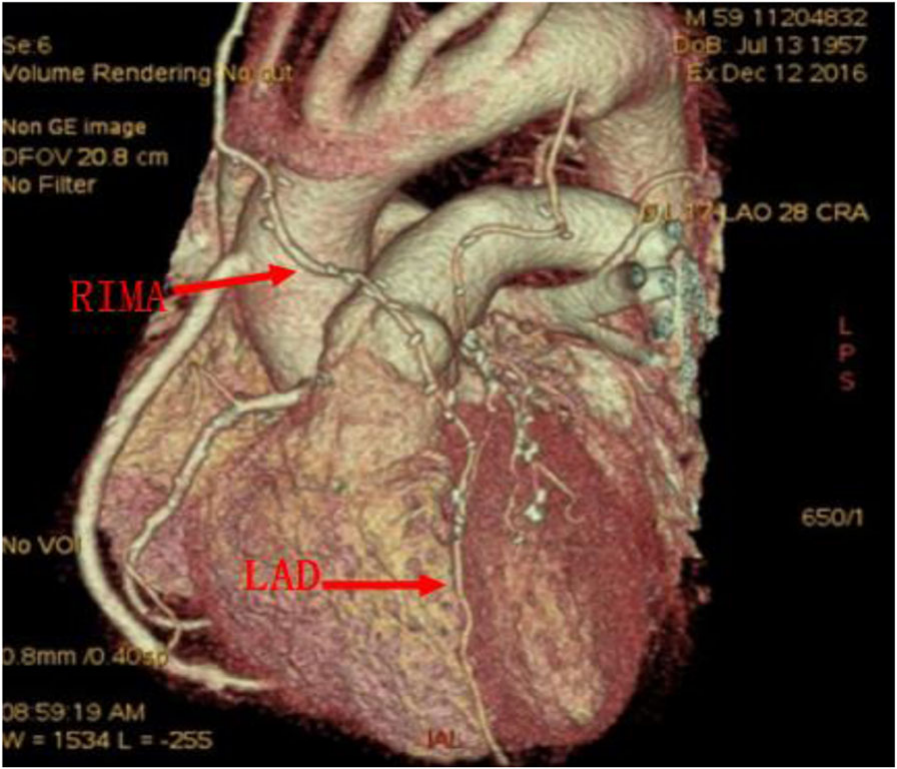
RIMA was in situ graft, and it was anastomosed to LAD. RIMA, right internal mammary artery; LAD, left anterior descending branch

**Fig. 3:**
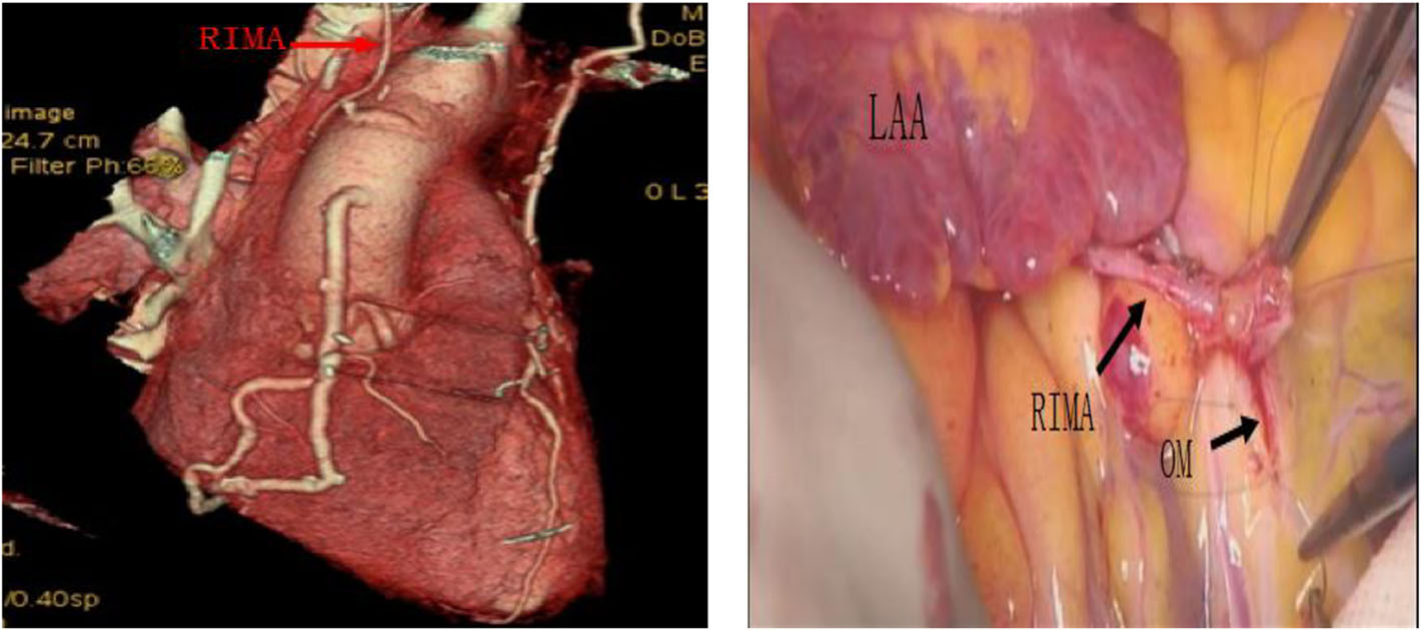
RIMA was in situ graft, and it was anastomosed to OM. RIMA, right internal mammary artery; LAA, left atrial appendage; OM, obtuse marginal branch

**Fig. 4:**
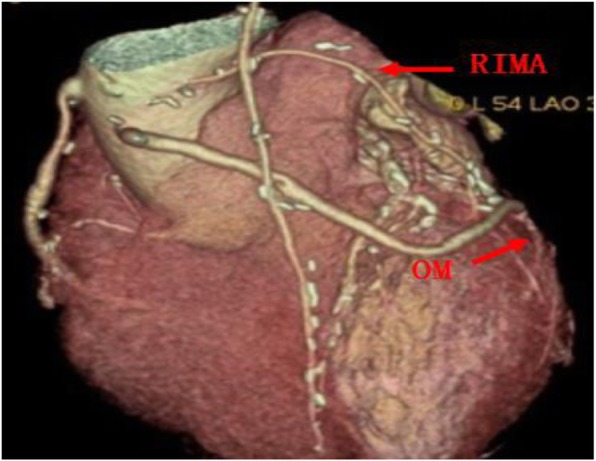
RIMA was free graft, and it was anastomosed to OM. RIMA, right internal mammary artery; OM, obtuse marginal branch

All the surgeries were performed by a single surgeon. The surgeries were performed via median sternotomy. The BIMA and great saphenous vein were harvested initially. In 39 patients of Group A and 25 of Group B, the internal mammary artery (IMA) was harvested with the skeletonization technique (Fig. 1). There were no significant differences in the IMA harvesting technique between these two groups (*P* = 0.94). After the IMA was freed completely, it was heparinised, and the distal end of the vessel was cut off. Then, it was wrapped in papaverine wet gauze for preservation. All of the surgeries were performed under cardiopulmonary bypass (CPB), mild hypothermia, and cardioplegic arrest. Ice water was placed in the pericardial cavity after cardiac arrest. The distal end of the graft was anastomosed initially, and then the proximal end of the graft was anastomosed after aortic unclamping. If the length of the IMA was insufficient, it was used as a free graft. The common grafting paths were as follows: the right internal mammary artery (RIMA) was anastomosed to the left anterior descending artery (LAD) across the midline (Fig. 2) or to the high obtuse marginal branch artery (OM) through the transverse sinus (Fig. 3); Sometimes, the RIMA was anastomosed to the right coronary artery (RCA); the left internal mammary artery (LIMA) was anastomosed to the diagonal branch artery, OM, or LAD; and the great saphenous vein was anastomosed to the posterior descending artery, left ventricular posterior artery, or OM. In Group A, all LIMAs were in situ grafts, while eight RIMAs were free grafts. Four of them were anastomosed to the LAD, and the other four to the high OM (Fig. 4). In Group B, all LIMAs were in situ grafts, while four RIMAs were free grafts; three of them were anastomosed to the high OM, and one to the RCA. The pathway of arterial grafts is shown in Table 1.

**Table 1:** Pathway and patency of arterial grafts

	Group A (*n* = 95)		Group B (*n* = 60)	
	Case	Occlusion (3 months post-operation)	Case	Occlusion (3 months post-operation)
LIMA				
LIMA-OM	51	1	41	–
LIMA-D	32	–	12	–
LIMA-LAD	12	–	7	–
RIMA				
RIMA-LAD	77	1	52	1
RIMA-OM	11	–	7	–
RIMA-RCA	7	–	1	–

*LIMA* left internal mammary artery, *OM* obtuse marginal branch artery, *D* diagonal branch artery, *LAD* left anterior descending artery, *RIMA* right internal mammary artery, *RCA* right coronary artery

#### Postoperative medication treatment

Patients without risk of bleeding were routinely treated with aspirin plus clopidogrel for 1 year after surgery, and then only with aspirin. Lipid-lowering, blood pressure control, heart rate control, and coronary artery dilatation were prescribed routinely unless they had contraindications. Echocardiography and coronary CTA were performed 3 months post-operation.

### Data collection and processing

Preoperative data collected included the sex ratio, age, body mass index (BMI), diabetes, hypertension, hyperlipidaemia, previous myocardial infraction, serum creatinine, peripheral artery stenosis, preoperative glycated haemoglobin A1c (HbA1c), cardiac function (New York Heart Association, NYHA, grade), left ventricular end-diastolic dimension (LVEDD), and left ventricular ejection fraction (LVEF). The operative time, aortic clamp time, CPB time, number of distal coronary anastomosis, flow rate and pulsatility index (PI) value of the LIMA and RIMA, and early postoperative complications were also collected. The flow rate and PI value of the IMA graft were measured by Veri Q system (Medistim, Sandakervn, Norway) under the condition of 60 mmHg mean arterial pressure and the anastomosis was completed. Patency of graft, LVEDD, and LVEF were measured in every patient with Doppler echocardiography and coronary artery CTA at 3 months after operation.

Mild sternal wound complications referred to delayed healing and sternal wound exudation without sternal loosening or wound infection. It could be cured by simple treatment, such as dressing change and local pressure without sternum re-fix and debridement. Severe sternal wound complications referred to sternum loosening or wound infection, which can only be cured by sternum re-fixation and wound debridement.

### Statistical analysis

Data were expressed as mean ± standard deviation (SD) or percentages of patients. Between-group comparisons of patient characteristics and intraoperative and postoperative variables were performed by the Chi-square test, Fisher’s exact test, Student’s t-test, or Mann-Whitney U test, as appropriate. All statistical data were analysed using SPSS 19.0 (IBM Inc., Armonk, NY), and *P* < 0.05 was considered statistically significant.

## Results

### Characteristics of the study population

There were 95 patients in Group A, the youngest of whom was 25 years old, with an average age of 52.08 ± 6.103 years. There were 60 patients in Group B, the oldest of whom was 75 years old, with an average age of 63.10 ± 3.112 years. The mean age of group B was significantly higher than that of Group A (*P* < 0.01). The incidence rate of diabetes and peripheral artery stenosis in group B was significantly higher than that in Group A (*P* = 0.002, 0.011). There were no significant differences in the sex ratio, BMI, hypertension, hyperlipidaemia, previous myocardial infraction, preoperative HbA1c, serum creatinine, preoperative cardiac function (NYHA), LVEDD, or LVEF between the groups. The characteristics of the study population are shown in Table 2.

**Table 2:** Characteristics of the Study Population

	Group A (n = 95)	Group B (*n* = 60)	*P* value
Female sex	13 (13.7%)	13 (21.7%)	0.195
Mean age ± SD, y	52.08 ± 6.103	63.10 ± 3.112	< 0.01
BMI (kg/m^2^)	26.12 ± 3.065	25.96 ± 2.656	0.745
Diabetes	28 (29.5%)	33 (55.0%)	0.002
Hypertension	55 (57.9%)	42 (70.0%)	0.129
Hyperlipidaemia	64 (67.4%)	36 (60.0%)	0.350
Previous myocardial infraction	35 (36.8%)	19 (31.7%)	0.510
Serum creatinine (μmol/L)	80.15 ± 13.40	82.31 ± 16.83	0.379
LVEDD (mm)	42.19 ± 4.879	48.88 ± 5.717	0.787
LVEF (%)	60.79 ± 5.630	60.77 ± 7.249	0.983
Cardiac function (NYHA)			0.188
I	20	9	–
II	65	41	–
III	10	10	–
Peripheral artery stenosis	15 (15.8%)	20 (33.3%)	0.011
Preoperative HbA1c(%)	6.15 ± 0.54	6.19 ± 0.51	0.673

*LVEDD* left ventricular end-diastolic dimension, *LVEF* left ventricular ejection fraction, *BMI* body mass index, *NYHA* New York Heart Association, *HbA1c* glycated haemoglobin A1c

### Intra-operative data

No significant difference could be found in the operative time, aortic clamp time, and CPB time between the groups (*P* > 0.05). There was no significant difference in the number of distal coronary anastomosis between Groups A and B (*P* = 0.896). The flow and PI value of LIMA were 28.63 ± 13.475 ml/min and 2.25 ± 0.459, while they were 30.12 ± 13.079 ml/min and 2.14 ± 0.475 for RIMA, respectively. There were also no significant differences in the flow and PI value of LIMA and RIMA between groups (*P* > 0.05). The intra-operative data of the study population are shown in Table 3.

**Table 3:** Intra-operative data of the Study Population

	Group A	Group B	*P* value
Operation time (h)	4.95 ± 0.622	4.92 ± 0.535	0.758
Aortic clamp time (min)	77.03 ± 18.135	77.47 ± 21.913	0.894
CPB time (min)	106.46 ± 23.573	107.88 ± 26.314	0.727
Number of distal coronary anastomosis	4.28 ± 0.821	4.27 ± 0.800	0.896
LIMA			
Flow (ml/min)	28.63 ± 13.475	28.25 ± 12.060	0.858
PI value	2.25 ± 0.459	2.18 ± 0.510	0.356
RIMA			
Flow (ml/min)	30.12 ± 13.079	26.80 ± 9.359	0.900
PI value	2.14 ± 0.475	2.16 ± 0.612	0.831

*CPB time* cardiopulmonary bypass time, *IMA* internal mammary artery, *PI* pulsatility index

### Short-term complications

After 3 months of follow-up, the operative mortality of both groups was zero, and there was no significant difference in bleeding requiring re-exploration, drainage volume on the day of operation, chylothorax, LVEF, and LVEDD between the groups (*P* > 0.05). There were two mild sternal wound complications in Group A but no severe sternal wound complication. There were three mild sternal wound complications and one severe sternal wound complication in Group B. There was no significant difference in the sternal wound complication between the groups (*P* > 0.05). After the 3-month follow-up, there were three venous graft occlusions in Group A and two in Group B. There were two arterials graft occlusions in Group A and one in Group B. There was no repeated CABG in both Groups A and B. The arterial graft occlusions in Group A were LIMA-OM and RIMA-LAD. The arterial graft occlusion in group B was RIMA-LAD. There was no significant difference in patency of grafts between the groups (*P* = 0.952, 0.847). The patency of arterial grafts is shown in Table 1. The other short-term complications of the study population are shown in Table 4.

**Table 4:** Short-term complications of the Study Population

	Group A (*n* = 95)	Group B (*n* = 60)	*P* value
Bleeding requiring re-exploration	1	0	0.425
Drainage volume on the day of operation (ml)	512.53 ± 246.71	493.67 ± 216.96	0.628
Chylothorax	3	4	0.306
Death	0	0	–
Sternal wound complication			
Mild	2	3	0.320
Severe	0	1	0.207
Patency of graft (3 months after operation)			
Venous graft occlusion	3	2	0.952
Arterial graft occlusion	2	1	0.847
Repeated CABG	0	0	–
Echocardiography characteristics (3-month after operation)			
LVEF (%)	60.60 ± 5.320	59.17 ± 5.901	0.119
LVEDD (mm)	46.62 ± 5.147	45.97 ± 4.902	0.434

*CABG* coronary artery bypass grafting

## Discussion

The main findings of this study were: 1) no significant differences were observed in the operation time, aortic clamp time, and CPB time between Groups A and B, so there was no significant difference in the difficulty of operation between these two groups; 2) there was no significant difference in the flow and PI index of arterial grafts of LIMA and RIMA between Groups A and B; thus, there was no significant difference in the immediate artery graft function between these two groups; and 3) there was no significant difference in the incidence rate of common complications, such as graft occlusion and sternal wound complications, between these two groups at 3 months post-operation.

### Operation experience

In this study, the skeletonized technique was preferred to be used in IMA harvesting and BIMA grafting was performed with extracorporeal circulation. In situ graft is recommended to reduce the risk of bleeding and the difficulty of operation, but if the length of arterial graft is insufficient, a free graft should be considered. Regarding the choice of graft for LAD, whether LIMA or RIMA, there is no literature to show the difference. We suggest that the length of graft and the extent of coronary artery lesions should be considered comprehensively in the selection of graft. Since the LIMA is closer to the left coronary system, it can provide a suitable length for the vascularization of the left coronary system; thus, the LIMA should be anastomosed to the circumflex artery or the diagonal branch artery if possible. In contrast, since RIMA is some distance from the left coronary system, it is often limited in length in the vascularization of the left coronary system; thus, it is mostly anastomosed to LAD, and sometimes it can be anastomosed to the high OM through the transverse sinus if necessary.

### Experience in preventing sternal complications

At present, the main factors which limit the application of BIMA grafting are poor sternal wound healing and deep sternal wound infection, especially for elderly women, patients with BMI > 40 kg/m^2^, patients with severe chronic obstructive respiratory dysfunction (COPD) or poor blood sugar control [10]. In this study, the incidence rate of mild sternal wound complications was 2.1% in Group A and 5% in Group B. The incidence rate of severe sternal wound complications was 0% in Group A and 1.7% in Group B. Although the prevalence of diabetes in Group B was higher than that in Group A, there was no significant difference in the incidence rate of sternal wound complications between these two groups, and the incidence rate of severe sternal wound complications was lower than 2.3–12.8% reported in the literature [11, 12]. Our experience in the prevention of sternal wound complications was as follows. 1) For diabetic patients, blood sugar should be strictly controlled during the perioperative period; particularly, preoperative HbA1c should be controlled below 6.5%. Previous studies have shown that elevated glycosylated haemoglobin was a high-risk factor for incision complications in cardiac surgery [13]. 2) The IMA harvested with the skeletonization technique was preferred as it inflicts little damage to the chest wall, and retains the branches of the IMA and internal mammary vein. It was beneficial to both the healing of sternal wound and venous reflux [14]. Therefore, in BIMA grafting, a number of guidelines have explicitly recommended the use of the skeletonization technique to harvest the IMA to reduce the incidence rate of sternal wound complications [15, 16]. Harvesting of the IMA using the skeletonization technique required precision and strictly abided by the principle of ‘no touch,’ paying careful attention to avoid arterial spasm and endothelial injury, as it was close to the main trunk of the IMA. 3) Commonly, the sternum was fixed by the “8-shaped method”. Five steel wires were used to fix the sternum in patients who weighed less than 50 kg. For patients who weighed more than 50 kg, one additional steel wire was added for every 10 kg increase in body weight. 4) Previous studies have shown that patients with a BMI > 40 kg/m^2^ had a high-risk for deep sternal wound complications in BIMA grafting [10], so attention should be paid to body weight. In this study, the maximum BMI of Group A was 38.06 kg/m^2^, with an average BMI of 26.12 ± 3.065 kg/m^2^, while that of Group B was 31.91 kg/m^2^, with an average BMI of 25.96 ± 2.656 kg/m^2^. The BMI of both groups was less than 40 kg/m^2^, and there was no significant difference between them.

This study has some limitations: 1) This was a retrospective, non-randomised study with no multivariate analysis therefore, a certain selection bias exists. Further prospective, randomised, large-scale, long-term studies with multivariate analysis are required to validate our findings. 2) The BMI of the study patients was lower than 40 kg/m^2^, so further studies are needed to confirm the role of BMI > 40 kg/m^2^ in the occurrence of sternal wound complications.

## Conclusions

BIMA grafting was safe and effective for older patients (60–75 years). Similar to younger patients (< 60 years), BIMA grafting in elderly patients (60–75 years) could also achieve a satisfactory short-term (3 months) result. Thus, advanced age (60–75 years old) should not be a contraindication for BIMA grafting.

## Data Availability

All data generated or analysed during this study are included in this published article and the supplementary information files.

## Abbreviations

BIMA: Bilateral internal mammary artery
BMI: Body mass index
CABG: Coronary artery bypass grafting
COPD: Chronic obstructive respiratory dysfunction
CPB: Cardiopulmonary bypass
CTA: Computed tomography angiography
HbA1c: Glycated haemoglobin A1c
IMA: Internal mammary artery
LAD: Left anterior descending branch
LIMA: Left internal mammary artery
LVEDD: Left ventricular end-diastolic dimension
LVEF: Left ventricular ejection fraction
NYHA: New York Heart Association
OM: Obtuse marginal branch
PI: Pulsatility index
RIMA: Right internal mammary artery
SD: Standard deviation
SIMA: Single internal mammary artery

## References

[1] Itoh S, Kimura N, Adachi H, Yamaguchi A (2016). Is bilateral internal mammary arterial grafting beneficial for patients aged 75 or older?. Circ J.

[2] Buxton BF, Shi WY, Tatoulis J, Fuller JA, Rosalion A, Hayward PA (2014). Total arterial revascularization with internal thoracic and radial artery grafts in triple-vessel coronary artery disease is associated with improved survival. J Thorac Cardiovasc Surg.

[3] Locker C, Schaff HV, Dearani JA, Joyce LD, Park SJ, Burkhart HM (2012). Multiple arterial grafts improve late survival of patients undergoing coronary artery bypass graft surgery: analysis of 8622 patients with multivessel disease. Circulation..

[4] Dimitrova KR, Hoffman DM, Geller CM, Dincheva G, Ko W, Tranbaugh RF (2012). Arterial grafts protect the native coronary vessels from atherosclerotic disease progression. Ann Thorac Surg.

[5] Kelly R, Buth KJ, Légaré JF (2012). Bilateral internal thoracic artery grafting is superior to other forms of multiple arterial grafting in providing survival benefit after coronary bypass surgery. J Thorac Cardiovasc Surg.

[6] Shi WY, Hayward PA, Tatoulis J, Rosalion A, Newcomb AE, Fuller JA (2015). Are all forms of total arterial revascularization equal? A comparison of single versus bilateral internal thoracic artery grafting strategies. J Thorac Cardiovasc Surg.

[7] Kieser TM, Lewin AM, Graham MM, Martin BJ, Galbraith PD, Rabi DM (2011). Outcomes associated with bilateral internal thoracic artery grafting: the importance of age. Ann Thorac Surg.

[8] Itagaki S, Cavallaro P, Adams D, Chikwe J (2013). Bilateral internal mammary artery grafts, mortality and morbidity: an analysis of 1526360 coronary bypass operations. Heart..

[9] Mohammadi S, Dagenais F, Doyle D, Mathieu P, Baillot R, Charbonneau E (2008). Age cut-off for the loss of benefit from bilateral internal thoracic artery grafting. Eur J Cardiothoracic Surg.

[10] Pettinari M, Sergeant P, Meuris B (2015). Bilateral internal thoracic artery grafting increases long-term survival in elderly patients. Eur J Cardiothorac Surg.

[11] Benedetto U, Altman DG, Gerry S, Gray A, Lees B, Pawlaczyk R (2016). Pedicled and skeletonized single and bilateral internal thoracic artery grafts and the incidence of sternal wound complications: insights from the arterial revascularization trial. J Thorac Cardiovasc Surg.

[12] Deutsch O, Gansera L, Wunderlich M, Eichinger W, Gansera B (2016). Does bilateral ITA grafting increase perioperative complications? Outcome of 6476 patients with bilateral versus 5020 patients with single ITA bypass. Thorac Cardiovasc Surg.

[13] Rollins KE, Varadhan KK, Dhatariya K, Lobo DN (2016). Systematic review of the impact of HbA1c on outcomes following surgery in patients with diabetes mellitus. Clin Nutr.

[14] Kamiya H, Akhyari P, Martens A, Karck M, Haverich A, Lichtenberg A (2008). Sternal microcirculation after skeletonized versus pedicled harvesting of the internal thoracic artery: a randomized study. J Thorac Cardiovasc Surg.

[15] Gatti G, Maschietto L, Morosin M, Russo M, Benussi B, Forti G (2017). Routine use of bilateral internal thoracic artery grafting in women: a risk factor analysis for poor outcomes. Cardiocasc revasc Med.

[16] Hashimoto M, Fukui T, Takanashi S (2015). Bilateral Internal Thoracic Artery Grafting: Is It Reasonable in Octogenarians?. Ann Thorac Cardiovasc Surg.

